# Supplementary material for: TAVI-PREP: A Deep Learning-Based Tool for Automated Measurements Extraction in TAVI Planning
Source: Diagnostics (Basel). 2023 Oct 11;13(20):3181. doi: 10.3390/diagnostics13203181 (PMC10606167; doi:10.3390/diagnostics13203181)
Supplement: Supplementary file 1 [file diagnostics-13-03181-s001.zip › diagnostics-2615802-supplementary.pdf]

Supplementary information

TAVI-PREP: A Deep Learning-Based Tool for Automated Measurements Extraction in TAVI Planning

Figure S1. Bland-Altman plot for aortic annulus

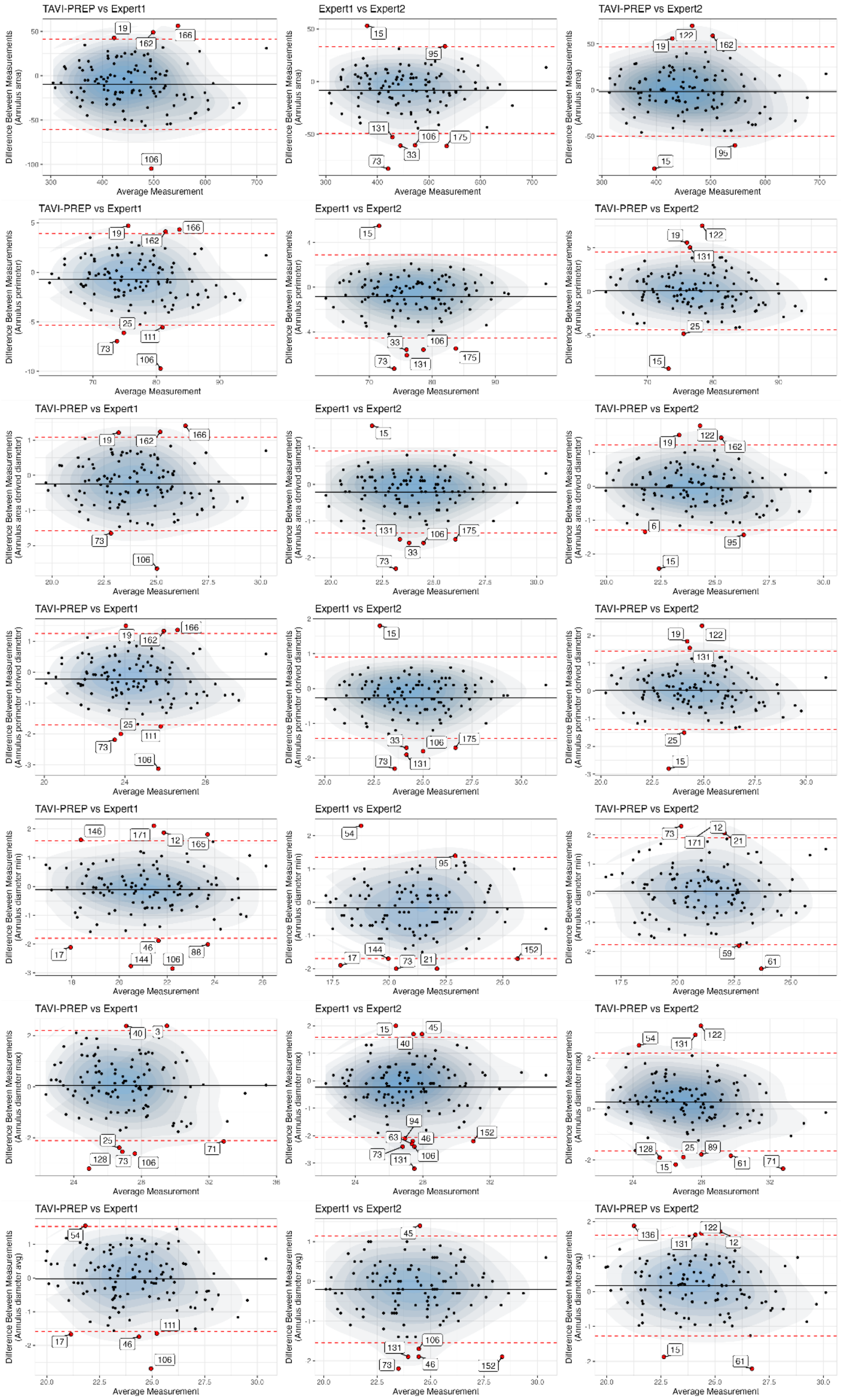

Figure S2. Bland-Altman plot for LVOT

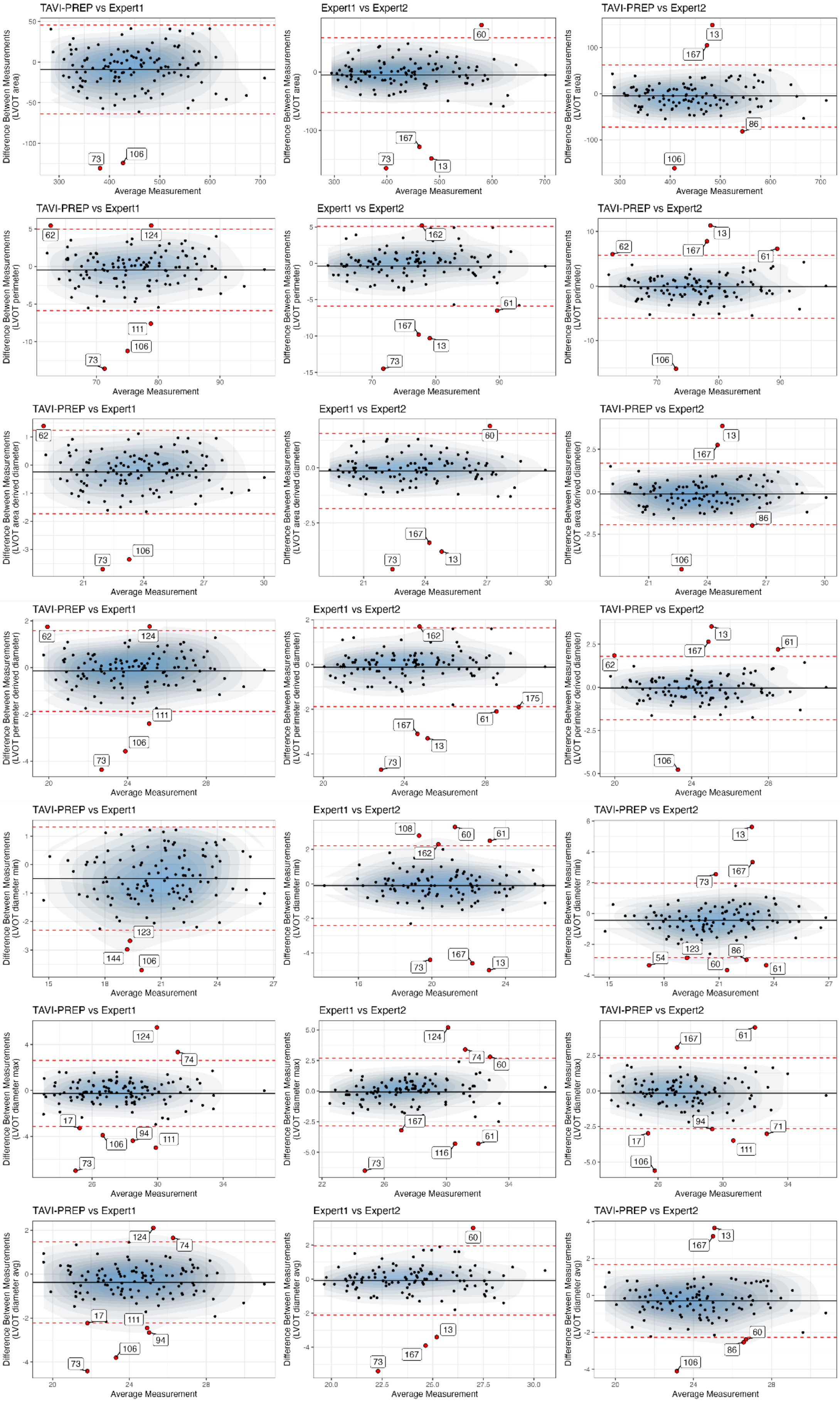

Figure S3. Bland-Altman plot for SNTJ

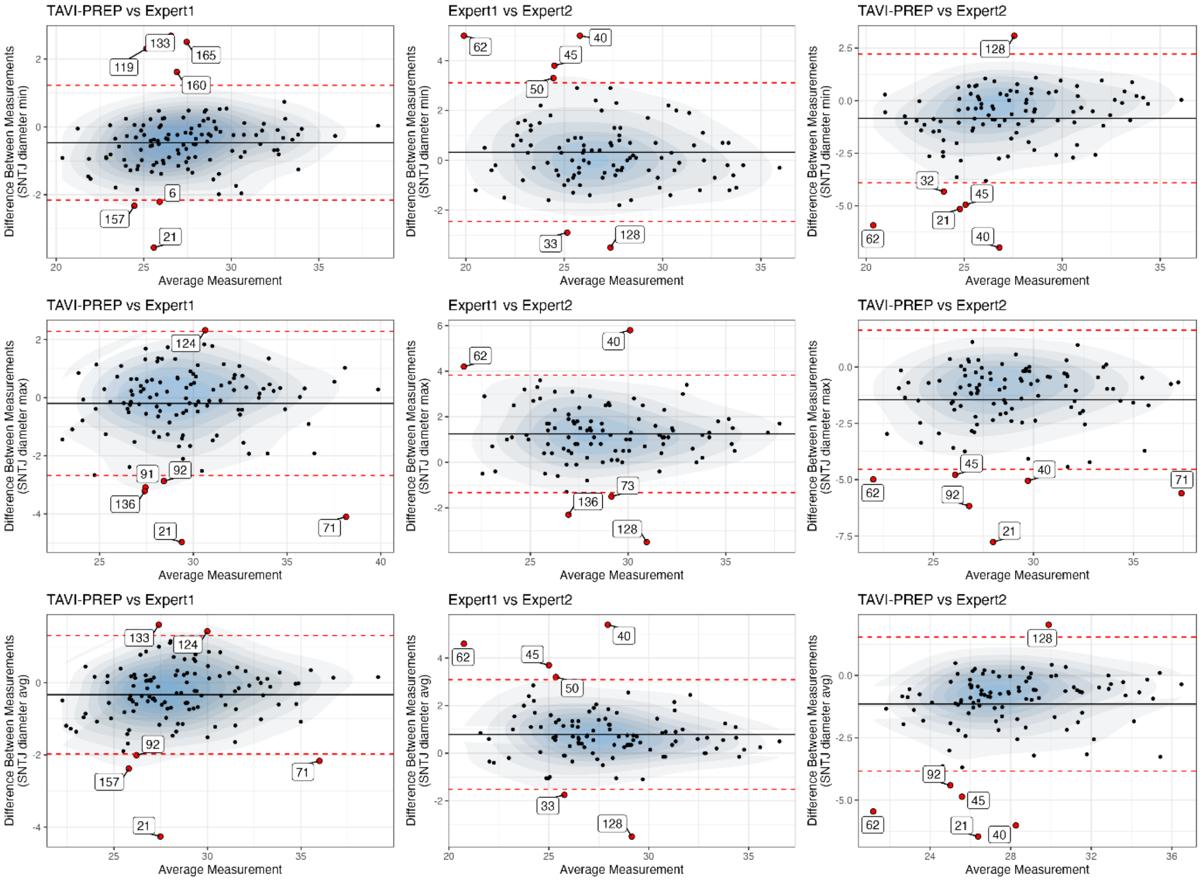

Figure S4. Bland-Altman plot for Sinus (RCC, LCC and NCC) and coronary heights (RCH, LCH)

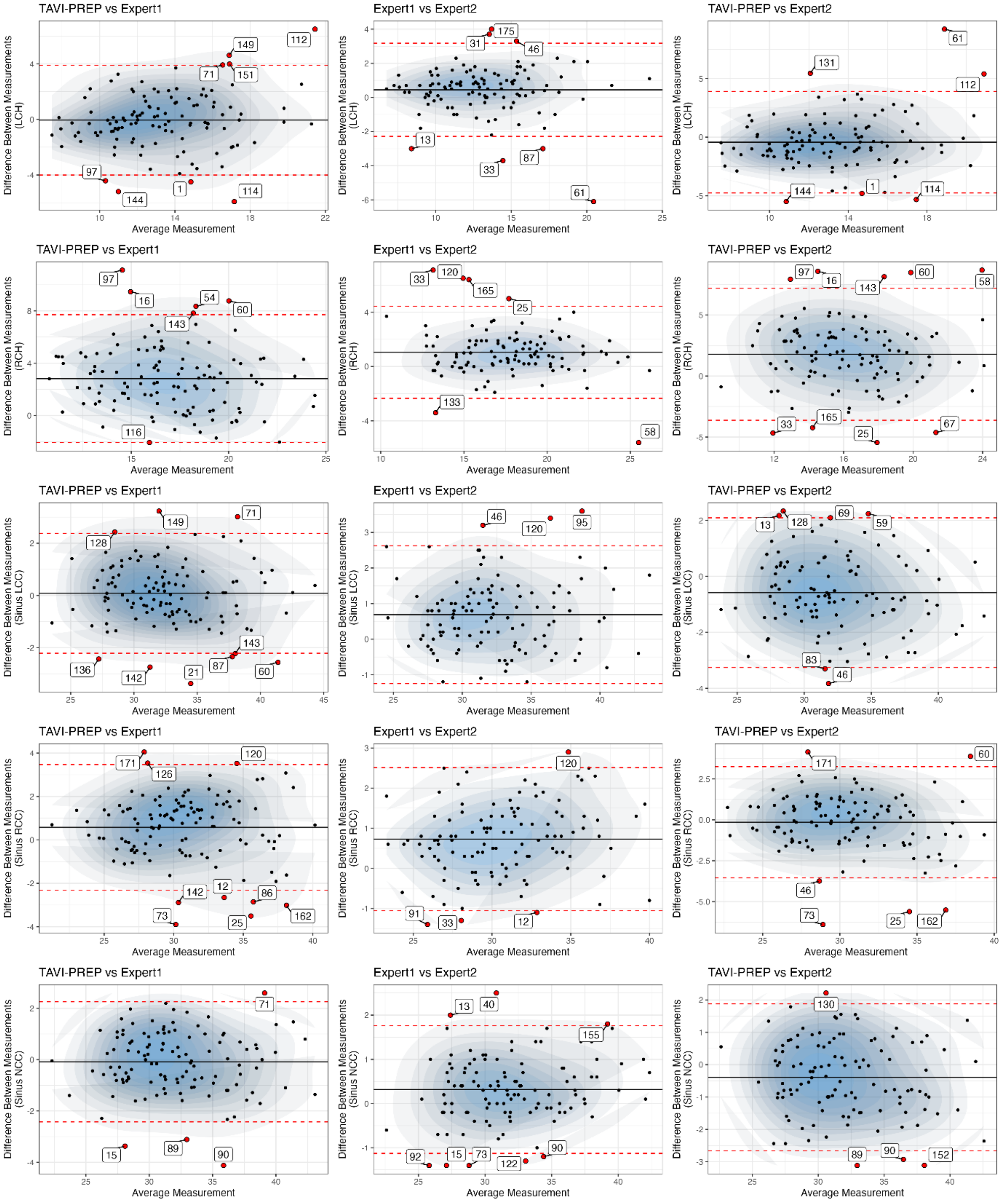

Table S1. TAVI-PREP vs Expert 1 correlation coefficients, absolute error and absolute relative error with their respective 95% confidence intervals

		Correlation	Correlation 95% lower	Correlation 95% upper	Absolute error	Absolute error 95% lower	Absolute error 95% higher	Absolute relative error	Absolute relative error 95% lower	Absolute relative error 95% higher	Absolute min error	Absolute max error
**Aortic annulus**	Area	0.949	0.931	0.962	-9.647	-60.650	41.357	4.879	4.204	5.553	-104.734	56.271
	Perimeter	0.939	0.918	0.955	-0.722	-5.345	3.902	2.493	2.125	2.861	-9.725	4.693
	Area derived diameter	0.948	0.930	0.962	-0.244	-1.575	1.087	2.421	2.095	2.746	-2.655	1.408
	Perimeter derived diameter	0.938	0.917	0.954	-0.230	-1.708	1.249	2.503	2.133	2.874	-3.124	1.498
	Diameter min	0.912	0.882	0.934	-0.104	-1.798	1.591	3.038	2.516	3.559	-2.853	2.109
	Diameter max	0.908	0.877	0.931	0.044	-2.114	2.203	3.147	2.676	3.619	-3.207	2.381
	Diameter avg	0.934	0.911	0.951	-0.029	-1.583	1.524	2.657	2.297	3.017	-2.687	1.546
**LVOT**	Area	0.951	0.934	0.963	-9.064	-63.762	45.634	5.046	4.034	6.058	-130.765	41.244
	Perimeter	0.933	0.910	0.950	-0.454	-5.877	4.968	2.602	2.075	3.128	-13.609	5.453
	Area derived diameter	0.948	0.931	0.962	-0.242	-1.729	1.244	2.478	2.010	2.946	-3.703	1.394
	Perimeter derived diameter	0.933	0.910	0.950	-0.142	-1.869	1.585	2.597	2.069	3.126	-4.363	1.762
	Diameter min	0.929	0.905	0.947	-0.492	-2.308	1.324	4.186	3.565	4.808	-3.708	1.218
	Diameter max	0.861	0.816	0.895	-0.260	-3.131	2.611	3.607	2.812	4.402	-6.983	5.485
	Diameter avg	0.924	0.898	0.943	-0.375	-2.223	1.472	3.045	2.448	3.642	-4.416	2.105
**SNTJ**	Diameter min	0.967	0.955	0.975	-0.465	-2.157	1.227	2.775	2.302	3.248	-3.555	2.688
	Diameter max	0.930	0.906	0.948	-0.198	-2.678	2.282	3.252	2.692	3.812	-4.968	2.320
	Diameter avg	0.968	0.957	0.976	-0.331	-1.974	1.311	2.421	1.998	2.845	-4.261	1.608
**Coronary**	LCH	0.799	0.736	0.848	-0.054	-4.002	3.894	11.604	9.681	13.526	-5.920	6.498
	RCH	0.718	0.635	0.784	2.822	-2.063	7.708	16.465	14.415	18.515	-2.069	11.120
**Sinus**	LCC	0.961	0.947	0.971	0.083	-2.214	2.379	2.824	2.424	3.225	-3.373	3.233
	RCC	0.929	0.905	0.948	0.578	-2.314	3.470	4.077	3.567	4.587	-3.897	4.046
	NCC	0.959	0.945	0.970	-0.085	-2.427	2.257	3.048	2.644	3.451	-4.125	2.597

Table S2. TAVI-PREP vs Expert 2 correlation coefficients, absolute error and absolute relative error with their respective 95% confidence intervals

		Correlation	Correlation 95% lower	Correlation 95% upper	Absolute error	Absolute error 95% lower	Absolute error 95% higher	Absolute relative error	Absolute relative error 95% lower	Absolute relative error 95% higher	Absolute min error	Absolute max error
**Aortic annulus**	Area	0.954	0.939	0.966	-1.695	-50.012	46.622	4.174	3.521	4.827	-85.300	69.604
	Perimeter	0.944	0.925	0.959	0.095	-4.344	4.534	2.216	1.860	2.571	-8.803	7.518
	Area derived diameter	0.954	0.938	0.966	-0.038	-1.299	1.223	2.062	1.743	2.381	-2.434	1.787
	Perimeter derived diameter	0.945	0.925	0.959	0.030	-1.378	1.439	2.224	1.872	2.575	-2.802	2.360
	Diameter min	0.897	0.863	0.923	0.067	-1.759	1.893	3.441	2.951	3.932	-2.583	2.309
	Diameter max	0.928	0.903	0.946	0.276	-1.652	2.204	2.858	2.421	3.296	-2.341	3.273
	Diameter avg	0.944	0.925	0.959	0.169	-1.270	1.608	2.495	2.156	2.833	-2.209	1.881
**LVOT**	Area	0.929	0.903	0.948	-4.828	-72.182	62.527	5.629	4.505	6.752	-161.598	149.041
	Perimeter	0.926	0.900	0.946	-0.112	-5.883	5.658	2.612	2.060	3.165	-15.153	11.089
	Area derived diameter	0.927	0.900	0.946	-0.131	-1.941	1.679	2.801	2.259	3.343	-4.554	3.870
	Perimeter derived diameter	0.926	0.900	0.946	-0.038	-1.875	1.798	2.619	2.070	3.168	-4.773	3.528
	Diameter min	0.881	0.840	0.912	-0.437	-2.850	1.977	4.537	3.684	5.390	-3.670	5.620
	Diameter max	0.893	0.856	0.921	-0.164	-2.650	2.322	3.238	2.603	3.874	-5.596	4.456
	Diameter avg	0.913	0.883	0.936	-0.303	-2.274	1.669	3.149	2.571	3.727	-4.102	3.650
**SNTJ**	Diameter min	0.901	0.865	0.928	-0.842	-3.902	2.218	4.878	3.730	6.026	-6.984	3.088
	Diameter max	0.895	0.857	0.923	-1.453	-4.540	1.634	5.748	4.596	6.900	-7.768	1.112
	Diameter avg	0.920	0.890	0.941	-1.148	-3.835	1.540	4.827	3.752	5.902	-6.461	2.034
**Coronary**	LCH	0.766	0.693	0.823	-0.429	-4.756	3.898	13.425	11.235	15.614	-5.497	9.214
	RCH	0.675	0.579	0.753	1.776	-3.630	7.183	15.334	13.287	17.380	-5.451	8.667
**Sinus**	LCC	0.946	0.927	0.960	-0.583	-3.255	2.090	3.867	3.360	4.374	-3.830	2.330
	RCC	0.898	0.864	0.925	-0.145	-3.544	3.254	4.331	3.640	5.022	-6.397	4.146
	NCC	0.961	0.947	0.971	-0.392	-2.661	1.877	3.161	2.748	3.574	-3.113	2.213

Table S3. Expert 1 vs Expert 2 correlation coefficients, absolute error and absolute relative error with their respective 95% confidence intervals

		Correlation	Correlation 95% lower	Correlation 95% upper	Absolute error	Absolute error 95% lower	Absolute error 95% higher	Absolute relative error	Absolute relative error 95% lower	Absolute relative error 95% higher	Absolute min error	Absolute max error
**Aortic annulus**	Area	0.965	0.953	0.974	-8.078	-49.298	33.142	3.748	3.071	4.425	-82.600	53.100
	Perimeter	0.959	0.944	0.970	-0.832	-4.557	2.894	1.966	1.609	2.322	-7.300	5.500
	Area derived diameter	0.963	0.949	0.972	-0.209	-1.326	0.907	1.853	1.517	2.188	-2.300	1.600
	Perimeter derived diameter	0.960	0.945	0.970	-0.266	-1.439	0.907	1.948	1.595	2.301	-2.300	1.800
	Diameter min	0.928	0.903	0.947	-0.172	-1.698	1.354	3.040	2.615	3.466	-2.000	2.300
	Diameter max	0.928	0.903	0.947	-0.238	-2.061	1.586	2.649	2.211	3.087	-3.200	2.000
	Diameter avg	0.947	0.929	0.961	-0.202	-1.543	1.140	2.295	1.942	2.647	-2.200	1.400
**LVOT**	Area	0.936	0.913	0.953	-5.224	-69.257	58.810	4.753	3.455	6.052	-164.900	80.600
	Perimeter	0.935	0.911	0.952	-0.389	-5.878	5.099	2.331	1.756	2.907	-14.500	5.200
	Area derived diameter	0.935	0.912	0.953	-0.139	-1.844	1.566	2.330	1.736	2.923	-4.600	1.900
	Perimeter derived diameter	0.933	0.910	0.951	-0.119	-1.882	1.644	2.347	1.766	2.928	-4.700	1.700
	Diameter min	0.893	0.855	0.921	-0.096	-2.409	2.216	3.780	2.936	4.624	-5.000	3.300
	Diameter max	0.875	0.832	0.908	-0.075	-2.847	2.698	3.298	2.534	4.061	-6.500	5.200
	Diameter avg	0.910	0.878	0.934	-0.084	-2.114	1.947	2.743	2.067	3.420	-5.400	3.000
**SNTJ**	Diameter min	0.919	0.889	0.941	0.327	-2.455	3.108	3.992	3.242	4.742	-3.500	5.000
	Diameter max	0.927	0.900	0.947	1.248	-1.331	3.827	5.078	4.372	5.784	-3.500	5.800
	Diameter avg	0.942	0.920	0.958	0.788	-1.516	3.091	3.791	3.111	4.472	-3.500	5.400
**Coronary**	LCH	0.915	0.886	0.937	0.448	-2.278	3.174	8.209	6.900	9.518	-6.100	4.000
	RCH	0.863	0.816	0.898	1.045	-2.345	4.436	8.090	6.595	9.585	-5.600	7.100
**Sinus**	LCC	0.970	0.960	0.978	0.689	-1.247	2.625	2.835	2.422	3.249	-1.200	3.600
	RCC	0.972	0.962	0.979	0.730	-1.053	2.513	3.046	2.673	3.418	-1.400	2.900
	NCC	0.984	0.979	0.989	0.318	-1.126	1.761	1.949	1.652	2.246	-1.400	2.500
